# Supplementary material for: The Cell–Cell Communication Signal Indole Controls the Physiology and Interspecies Communication of Acinetobacter baumannii
Source: Microbiol Spectr. 2022 Jul 6;10(4):e01027-22. doi: 10.1128/spectrum.01027-22 (PMC9431217; doi:10.1128/spectrum.01027-22)
Supplement: Supplemental file 1 — Supplemental material. Download spectrum.01027-22-s0001.pdf, PDF file, 1.7 MB [file spectrum.01027-22-s0001.pdf]

Supplementary Information

The cell-cell communication signal indole controls the physiology and interspecies communication of *Acinetobacter baumannii*

Binbin Cui[1#], Xiayu Chen[1#], Quan Guo[1], Shihao Song[1], Mingfang Wang[1], Jingyun Liu[2] and Yinyue Deng[1*]

[1]*School of Pharmaceutical Sciences (Shenzhen), Shenzhen Campus of Sun Yat-sen University, Sun Yat-sen University, Shenzhen 518107, China*

[2]*Department of Stomatology, Zhengzhou Shuqing Medical College, Zhenzhou 450064, China*

[#]These authors contributed equally to this work.

*To whom correspondence may be addressed. Email: Yinyue Deng: dengyle@mail.sysu.edu.cn

Running title: Indole controls the physiology of *A. baumannii*

This file includes:

Supplementary Methods

Supplementary Figures S1 to S10

Supplementary Tables S1 to S6

SUPPLEMENTARY METHODS

Phenotypic analysis. Biofilm formation was quantified in 96-well polystyrene plates as mentioned earlier (1). A single colony of each strain was inoculated and grown overnight at 37°C with agitation in LB medium. Bacterial cells were diluted to an optical density at 600 nm (OD_{600}) of 0.1 with LB medium and added to 96-well polystyrene plates. After incubation at 37°C for 24 h, the cells were stained with 0.1% crystal violet (CV) for 30 min. The cultures were poured out, stained with crystal violet for 15 min, and washed several times with ddH_2O before adding 95% ethanol. The optical density at 590 nm (OD_{590}) of the solution was measured to quantify biofilm formation.

Motility was measured on semisolid agar. Bacteria were inoculated on the center of plates containing 1% tryptone, 0.5% NaCl and 0.3% agar. The plates were incubated at 37°C for 8 h before the diameter of the movement trace was measured.

Cytotoxicity was assessed by measuring the release of lactate dehydrogenase (LDH) from A549 cells. The A549 cells were grown in DMEM supplemented with 10% fetal bovine serum (FBS) in 96-well tissue culture plates at 1×10^5 cells/well. Confluent A549 cells were washed and incubated with DMEM containing 1% FBS before infection. Bacterial cells were grown in LB medium at 37°C overnight, centrifuged and resuspended in culture medium. A549 cells were infected with bacterial cells at 10^9 CFU/mL for 8 h. After incubation, the LDH in the supernatant was measured, and cytotoxicity was calculated relative to the uninfected control group.

Growth rate analysis. Bacterial strains were cultured in LB medium for 18-24 h. Then, the cultures were washed twice in fresh LB medium or MP minimal medium and inoculated into

fresh media to an OD_{600} of 0.01 (LB medium) or 0.1 (MP minimal medium). Growth curves were constructed in triplicate by incubating the cells for 24 h at 37°C with shaking at 200 rpm. Bacterial growth was monitored by measuring the OD_{600} values of the culture. Blank LB medium or MP minimal medium was used as the negative control.

Quantitative analysis of QS signal production. Bacterial cells were grown in LB medium overnight with agitation at 37°C. One liter of culture supernatant was collected by centrifugation and extracted with an equal volume of ethyl acetate. The crude extract (organic phase) was dried using a rotary evaporator and dissolved in methanol. All of the above samples were kept at 4°C until analysis. Ultrahigh-performance liquid chromatography-electrospray ionization tandem mass spectrometry (UHPLC-ESI-MS/MS) was performed in a Shimadzu LC-30A UHPLC system with a Waters C_{18} column (1.8 μm, 150 × 2.1 mm) and a Shimadzu 8060 QQQ-MS mass spectrometer with an ESI source interface. The mass spectrometer was operated in positive-ion mode. The mobile phase was prepared as 0.1% formic acid/water and acetonitrile.

Quantitative analysis of extracellular indole. Bacterial strains were inoculated and grown overnight at 37°C with shaking in LB medium. The cultures were then diluted to a starting OD_{600} = 0.1 in fresh medium and cultured overnight at 37°C with shaking at 200 rpm. The culture supernatant was collected by centrifugation and extracted with equal volume of ethyl acetate/ dichloromethane (v:v=4:1). Crude extract was dried in a rotary evaporator and dissolved in methanol. All the above samples were kept at 4°C before analysis. The detection method was based on the previous description and has been slightly modified (2). UHPLC-ESI-MS/MS was performed in a Shimadzu LC-30A UHPLC system with a Waters

C_{18} column (1.8 μm, 150 × 2.1 mm) and a Shimadzu 8060 QQQ-MS mass spectrometer with an ESI source interface. The mass spectrometer was operated in positive-ion mode. The mobile phase was prepared as 0.1% formic acid/water and 0.1% formic acid/ACN.

Quantitative RT-PCR analysis. Bacterial cells were cultured to 2 × 10^9 cfu/mL and then harvested. RNA was isolated using an Eastep Super Total RNA Extraction Kit (Promega, Madison, USA). cDNA synthesis and RT-qPCR analysis were performed with ChamQ™ Universal SYBR qPCR Master Mix (Vazyme, Nanjing, China) according to the manufacturer's instructions in a 7300 Plus Quantitative Real-Time PCR System (Thermo Fisher Scientific, New York, USA).

Expression levels of target genes were normalized to the level of the 16S RNA transcript for each experiment. The relative expression levels of the target genes were calculated using the comparative CT ($2^{-\Delta\Delta CT}$) method (3).

RNA-seq analysis. Double-stranded cDNA synthesis and high-throughput RNA-seq were performed as previously described (4). Three biological replicates were sequenced for each strain. Trimmed sequence reads were aligned to the *A. baumannii* ATCC17978 genome sequence using Bowtie2-2.2.3 (5), and normalized read counts were compared by HTSeq v0.6.1 as previously described (6). For each duplicate sample, between 7.3 million and 10.2 million sequence reads were uniquely mapped to the *A. baumannii* ATCC17978 genome sequence. Differentially expressed genes were identified as those with a high expression level ($\log_2 \geq 1.0$) across all replicates with a false discovery rate (FDR) of < 0.01.

Mouse airway infection assays. Male BALB/c mice aged 6-8 weeks were purchased from Guangdong Medical Experimental Animal Center. Before the experiment, the mice were

allowed to adapt to a standard diet for 7 d in the experimental animal center of South China Agricultural University. All the mice were allowed free access to food and water. The freshly cultured strains were inoculated into 10 mL of LB culture medium and cultured overnight at 37°C with shaking at 200 rpm. The cultures were centrifuged to collect cells, which were resuspended and washed with 1 × PBS twice and finally resuspended in PBS to an OD_{600} of 1.0.

Male BALB/c mice were randomly divided into 4 groups. Each group consisted of 6 mice, which were inhaled 10 μL of bacterial solution via the nasal cavity. PBS was used for the blank control group. At 7 d postinfection, 6 mice in each group were euthanized, and the lungs were harvested and processed for histopathological analysis. Tissues were fixed in 10% formaldehyde, embedded in paraffin, sectioned, stained with hematoxylin-eosin (HE), and examined by light microscopy. The micrographs of the HE-stained lung tissue were reviewed by a pathologist.

SUPPLEMENTARY FIGURES AND TABLES

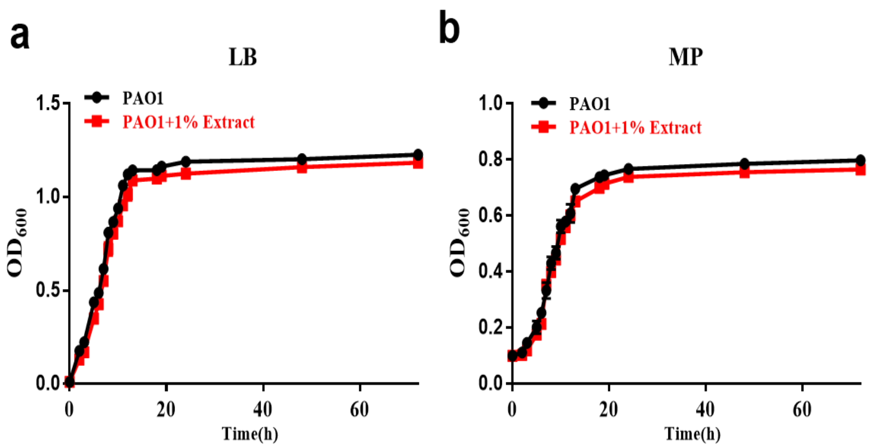

SUPPLEMENTARY FIGURE 1 Analysis of the growth of the PAO1 wild-type strain in the absence or presence of *A. baumannii* extract. The experiment was started at an initial OD_{600} of 0.01 in LB medium (a) and 0.1 in MP minimal medium (b) at 37°C with three replicates in a low-intensity shaking model using the Bioscreen-C automated growth curve analysis system. Extract was dissolved in methanol, and the same volume of methanol used as the solvent for the compounds was used as a control. The data are the means ± standard deviations of three independent experiments.

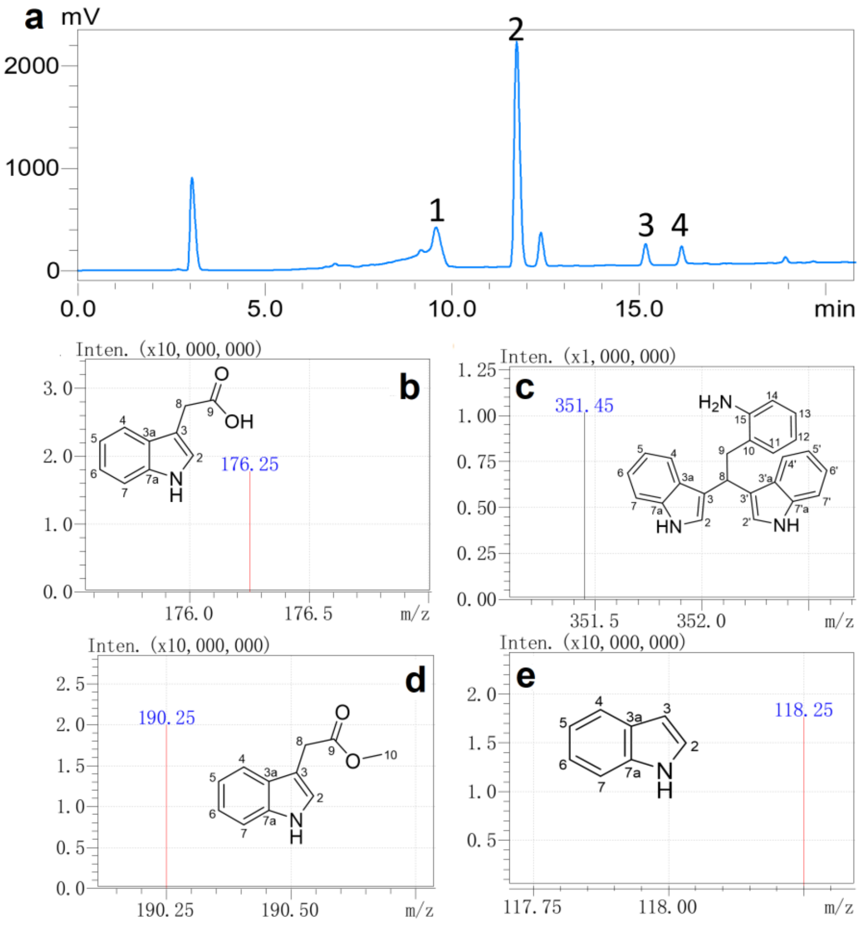

SUPPLEMENTARY FIGURE 2 Active compounds isolated from *A. baumannii* extract.

(a) Chromatogram of bioactive fractions of *A. baumannii* extracts. Peak 1: IAA; peak 2: di-IEA; peak 3: MIA; peak 4: indole. ESI-MS spectra and structure of IAA (b), di-IEA (c), MIA (d) and indole (e).

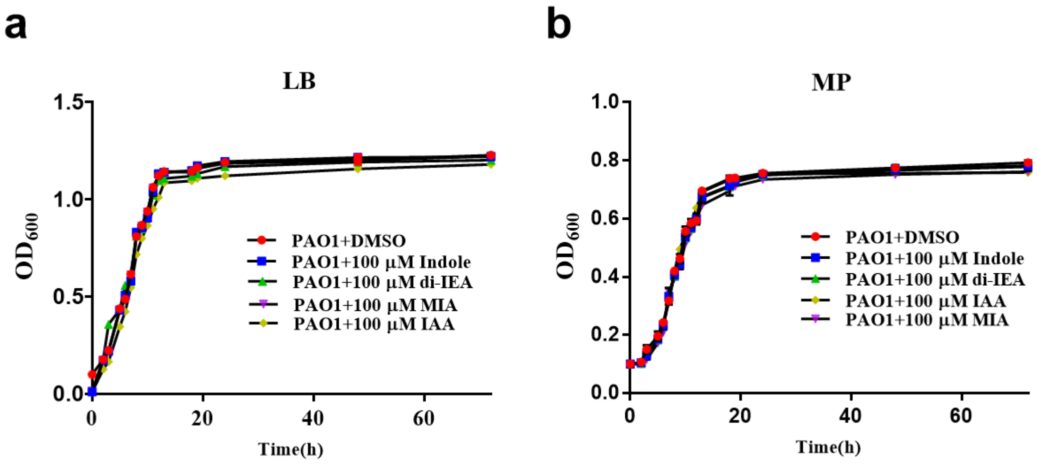

SUPPLEMENTARY FIGURE 3 Analysis of the growth curve of the PAO1 wild-type strain in the absence and presence of 100 *μ*M indole and 100 *μ*M indole derivatives. The experiment was started at an initial OD_{600} of 0.01 in LB medium (a) and 0.1 in MP minimal medium (b) at 37°C with three replicates in a low-intensity shaking model using the Bioscreen-C automated growth curve analysis system. Compounds were dissolved in DMSO, and the same volume of DMSO used as the solvent for the compounds was used as a control. The data are the means ± standard deviations of three independent experiments.

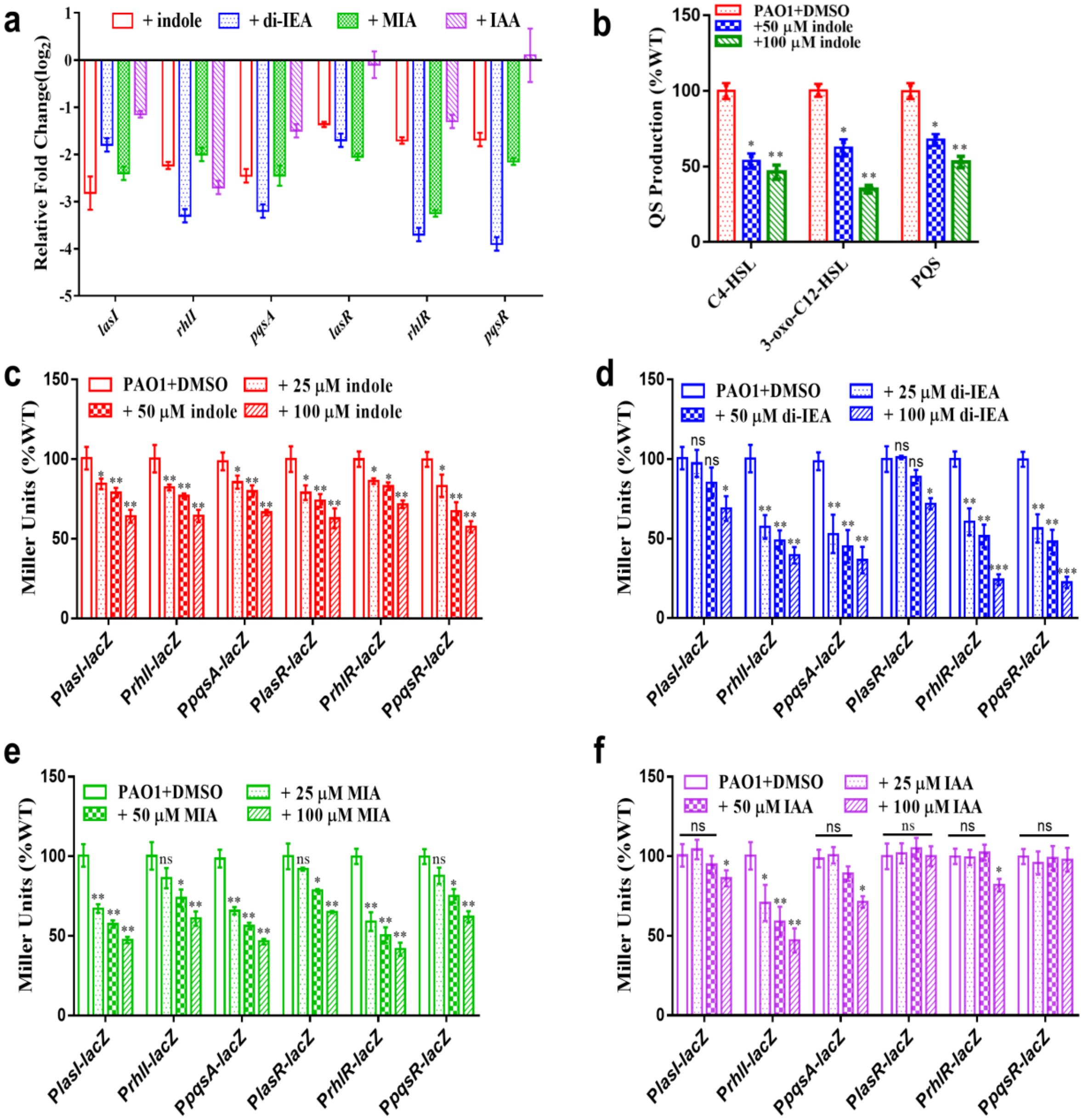

SUPPLEMENTARY FIGURE 4 Effects of indole and its derivatives on the QS and T3SS systems of *P. aeruginosa*. (a) The effects of 100 μM indole, 100 μM di-IEA, 100 μM and 100 μM IAA on the expression of signal synthase-encoding genes and regulator-encoding genes of QS systems were evaluated by RT-qPCR (OD_{600} = 1.0). (b) QS signal production in wild-type PAO1 in the absence or presence of indole. The amount of each signal in the PAO1 wild-type strain was arbitrarily defined as 100% and used to normalize the amount of

that signal in the PAO1 strain supplemented with indole. Inhibitory effect of indole (c), di-IEA (d), MIA (e) and IAA (f) on the expression of the QS systems, as determined by using P*lasI*-*lacZ*, P*rhlI*-*lacZ*, P*pqsA*-*lacZ*, P*lasR*-*lacZ*, P*rhlR*-*lacZ* and P*pqsR*-*lacZ* transcriptional fusions reporter strains (OD_{600} = 3.0). The β-galactosidase activity of each promoter-*lacZ* in the PAO1 wild-type strain was arbitrarily defined as 100% and used to normalize the β-galactosidase activity of each promoter-*lacZ* in the PAO1 strain supplemented with the compounds. Compounds were dissolved in DMSO, and the same volume of DMSO used as the solvent for the compounds was used as a control. The data are the means ± standard deviations of three independent experiments. The significance was determined by twone-way ANOVA (*$p < 0.05$; **$p < 0.01$; ***$p < 0.001$; ns = no significance).

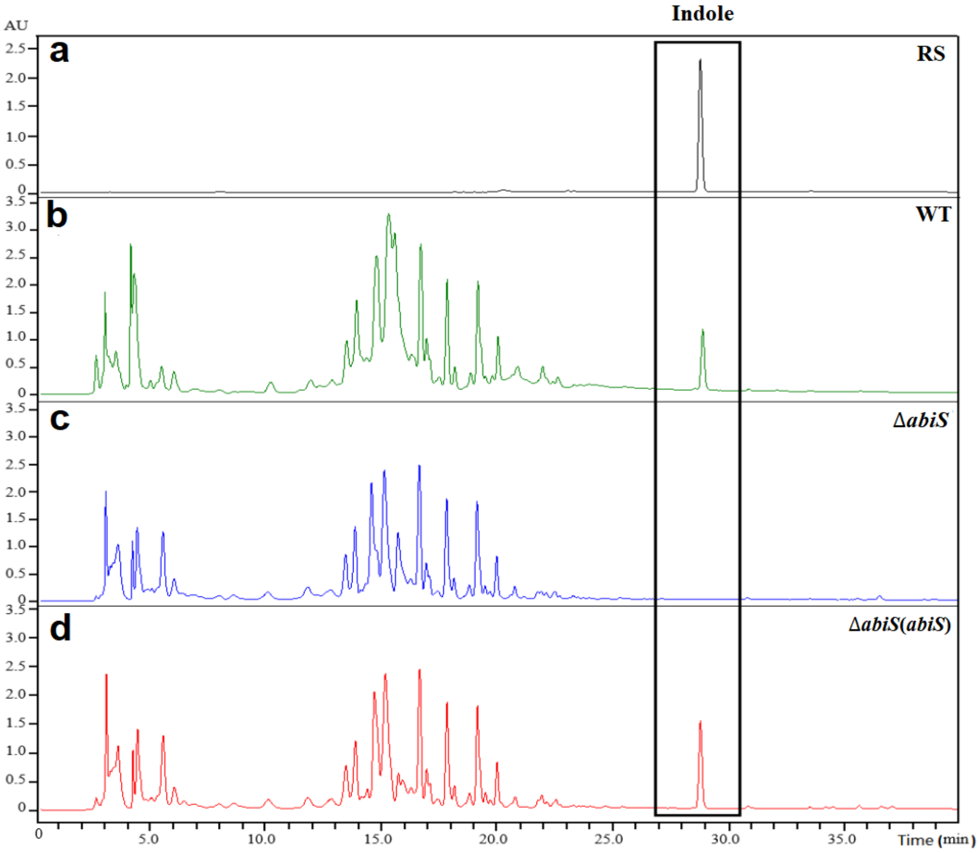

SUPPLEMENTARY FIGURE 5 HPLC chromatogram of indole. Chromatograms of the (a) indole reference substance (RS) sample, (b) *A. baumannii* WT culture extract, (c) Δ*abiS* culture extract, and (d) Δ*abiS*(*abiS*) culture extract. The retention time of the indole chromatographic peak was 28.89 min.

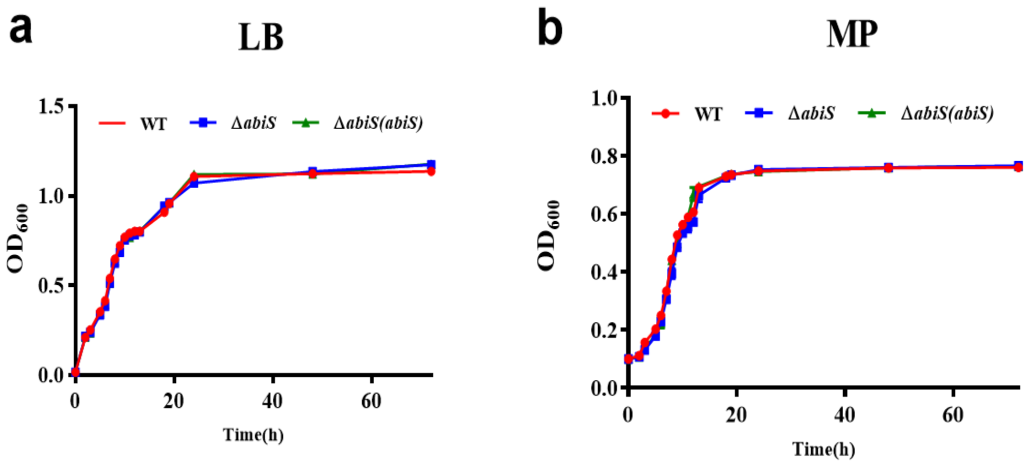

SUPPLEMENTARY FIGURE 6 Effect of *abiS* on the growth of *A. baumannii* ATCC17978 in LB medium (a) and MP minimal medium (b). The cells were inoculated at 37°C with three replicates in a low-intensity shaking model using the Bioscreen-C automated growth curve analysis system. The experiment was started at an initial OD_{600} of 0.01 in LB medium and 0.1 in MP minimal medium. The data are the means ± standard deviations of three independent experiments.

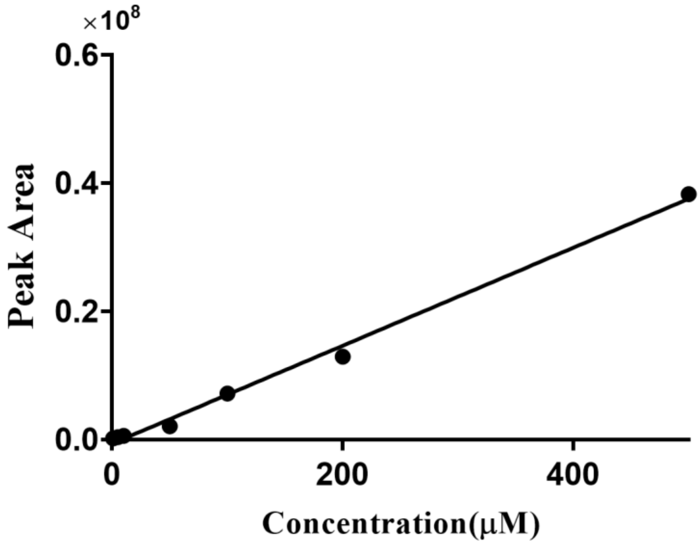

SUPPLEMENTARY FIGURE 7 Calibration curves were made by plotting the peak area (*Y*) versus the concentrations (*X*, *μ*M) of the standard solutions of indole. The regression equation of indole was $Y = 0.0007639X - 0.006467$, and the linear $R^2 = 0.9948$ (n = 7).

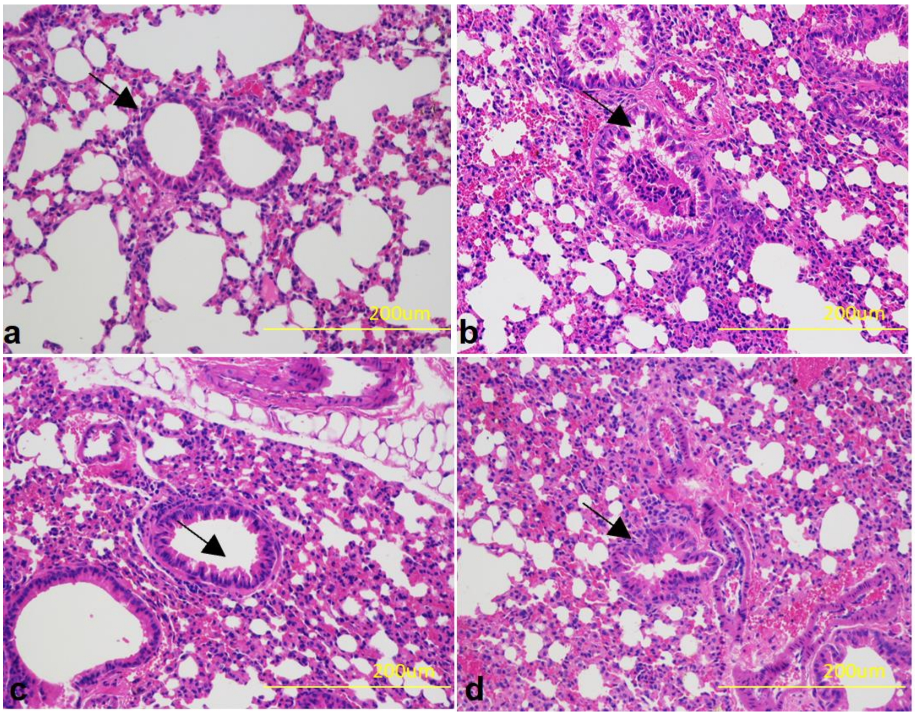

SUPPLEMENTARY FIGURE 8 Influences of AbiS on the pathogenicity of *A. baumannii* in a mouse infection model. Histological changes in lung tissue infected for 7 d assessed by HE (magnification, × 200). (a) Control group: healthy mice with PBS processing. (b) WT processing group. (c) Δ*abiS* processing group. (d) Δ*abiS* (*abiS*) processing group.

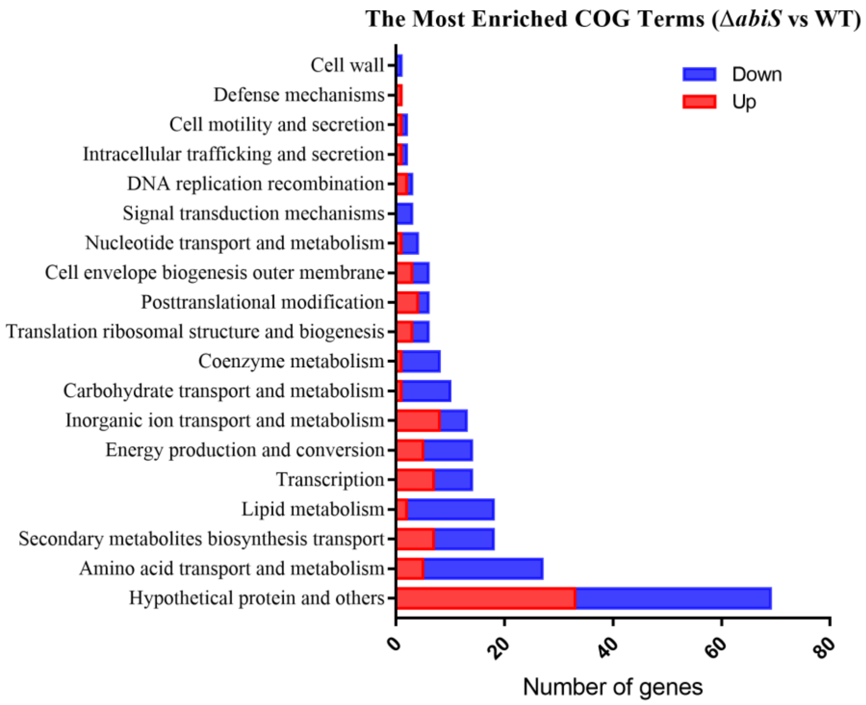

SUPPLEMENTARY FIGURE 9 Differential gene expression profiles between the *A. baumannii* ATCC17978 *abiS* mutant strain and the wild-type strain as measured by RNA-seq (Log_2 fold-change ≥ 1.0). COG term enrichment analysis of differentially expressed genes between the *abiS* mutant strain and the wild-type strain.

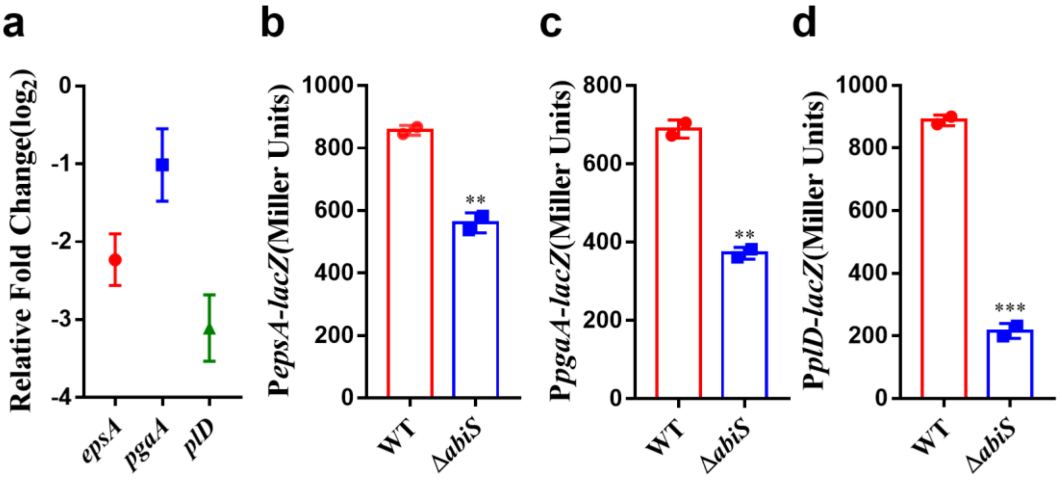

SUPPLEMENTARY FIGURE 10 Effects of AbiS on the expression levels of virulence-related genes of *A. baumannii*. (a) The expression levels of the *epsA*, *pgaA* and *plD* genes in the *abiS* mutant strain compared to the wild-type strain were evaluated by RT-qPCR. The expression of *epsA* (b), *pgaA* (c) and *plD* (d) was evaluated by assessing the β-galactosidase activity of the promoter-*lacZ* transcriptional fusions in the ATCC17978 wild-type and *abiS* mutant strains. The data are the means ± standard deviations of three independent experiments. The significance was determined by unpaired *t* test (*$p < 0.05$; **$p < 0.01$; ***$p < 0.001$; ns = no significance).

SUPPLEMENTARY TABLE 1 Analysis of the homologs of AbiS in various bacterial species

Scientific Name	Per. Ident (%)	Accession
Acidovorax		
A. delafieldii	50.51	WP_146870041.1
A. facilis	50.85	WP_182118373.1
A. kalamii	51.19	WP_094288937.1
A. radicis	50.17	WP_167539790.1
A. soli	50.00	WP_184858798.1
Acinetobacter		
A. albensis	62.86	WP_193889523.1
A. baylyi	56.76	WP_004923853.1
A. beijerinckii	50.50	WP_005053624.1
A. bouvetii	68.87	WP_174558281.1
A. calcoaceticus	95.85	MBP1058051.1
A. celticus	67.30	WP_068889112.1
A. guillouiae	72.17	WP_209828209.1
A. indicus	71.02	WP_104490279.1
A. johnsonii	65.29	HAE64211.1
A. junii	70.29	WP_151816724.1
A. kookii	68.24	WP_092820167.1
A. kyonggiensis	70.32	WP_092689631.1
A. lactucae	95.53	WP_125738878.1
A. lanii	68.47	WP_166368253.1
A. puyangensis	68.03	WP_097078814.1
A. radioresistens	70.97	WP_111282465.1
A. schindleri	73.25	WP_004809936.1
A. seifertii	94.57	WP_081401410.1
A. seohaensis	72.93	GIT82926.1
A. shaoyimingii	63.40	WP_166011152.1
A. terrae	68.47	WP_171534095.1
A. terrestris	67.20	WP_171537998.1
A. tjernbergiae	70.65	WP_023274803.1
A. towneri	74.52	HHW52401.1
Alcanivorax		
A. hongdengensis	53.87	WP_008930191.1
A. jadensis	50.51	WP_052042766.1
A. nanhaiticus	50.85	WP_035229980.1
A. sediminis	51.88	WP_153500944.1
Alkanindiges		
A. hydrocarboniclasticus	55.49	ONG42222.1
A. illinoisensis	55.80	TEU29427.1
Aquabacterium		
A. fontiphilum	51.69	WP_161650590.1

A. olei	54.11	WP_109038120.1
Duganella		
D. callida	51.60	WP_135201243.1
D. ginsengisoli	50.69	WP_155441037.1
D. levis	51.57	WP_161057701.1
D. rivi	50.17	WP_154356859.1
Janthinobacterium		
J. agaricidamnosum	50.00	WP_038491651.1
J. violaceinigrum	50.34	WP_152285270.1
Marinobacter		
M. adhaerens	50.69	HBF94108.1
M. alexandrii	50.00	WP_138442180.1
M. algicola	50.34	WP_007153457.1
M. antarcticus	51.22	WP_072794777.1
M. salsuginis	51.38	WP_153634906.1
M. similis	50.00	AHI29058.1
M. subterrani	50.34	WP_048497712.1
Noviherbaspirillum		
N. autotrophicum	50.68	WP_040039514.1
N. galbum	50.00	WP_163960454.1
N. massiliense	50.17	WP_019140047.1
Pseudomonas		
P. aestusnigri	52.70	WP_088276244.1
P. alcaligenes	52.70	WP_076578620.1
P. alcaliphila	50.98	WP_074678193.1
P. anguilliseptica	51.50	WP_090386409.1
P. azotifigens	53.45	WP_181072163.1
P. laoshanensis	50.67	WP_149333116.1
P. leptonychotis	51.50	WP_136664772.1
P. litoralis	50.16	WP_090274710.1
P. urumqiensis	54.48	WP_120997780.1
P. viridiflava	51.00	WP_122496341.1
P. xanthomarina	54.98	WP_125877688.1
Undibacterium		
U. aquatile	52.40	WP_190477350.1
U. crateris	51.03	NDI86481.1
U. oligocarboniphilum	51.03	WP_176804112.1
U. pigrum	51.71	WP_110257669.1
U. piscinae	50.17	QJQ07049.1
U. terreum	51.20	WP_188565314.1
Vibrio		
V. aestuarianus	51.19	WP_168524545.1
V. metoecus	50.68	WP_055050444.1
V. ostreicida	51.33	WP_083949304.1

V. ouci	50.50	WP_134834360.1
V. toranzoniae	50.00	WP_202601757.1
**Others**		
Abscondita terminalis	57.32	KAF5295391.1
Aquirhabdus parva	54.70	WP_114898035.1
Bermanella marisrubri	52.19	WP_007018521.1
Caldimonas taiwanensis	50.00	WP_157451265.1
Fluviicoccus keumensis	51.84	WP_130415403.1
Grimontia celer	50.00	WP_062665096.1
Kaistia algarum	54.55	WP_207778865.1
Klebsiella pneumoniae	99.45	WP_159413373.1
Mangrovitalea sediminis	50.34	WP_097461948.1
Oleiphilus messinensis	51.85	WP_087464611.1
Oppiella nova	70.32	CAD7636144.1
Polaromonas glacialis	50.34	WP_036772416.1
Rugamonas rubra	50.00	WP_093388806.1
Salmonella enterica	65.67	EAO7613976.1

SUPPLEMENTARY TABLE 2 Analysis of the homologs of TnaA in various bacterial species

Scientific Name	Per. Ident (%)	Accession
Acinetobacter		
A. pittii	100.00	WP_068547491.1
A. indolicus	89.38	WP_162856605.1
Aeromonas		
A. aquatica	84.50	WP_033132533.1
A. caviae	84.93	WP_201891148.1
A. diversa	82.80	WP_005350324.1
A. encheleia	84.29	WP_042651772.1
A. enteropelogenes	84.50	WP_223379182.1
A. eucrenophila	84.93	WP_042642663.1
A. hydrophila	85.35	WP_025328808.1
A. lusitana	84.93	WP_100860904.1
A. media	85.35	WP_205642793.1
A. media WS	85.14	AHX63314.1
A. rivipollensis	85.35	WP_163155242.1
A. sanarellii	85.14	WP_042074029.1
A. taiwanensis	84.93	WP_043763952.1
A. tecta	84.93	WP_050720465.1
Citrobacter		
C. amalonaticus	93.81	SUX72002.1
C. braakii	91.72	WP_080860469.1
C. freundii complex	91.72	WP_048220613.1
C. sedlakii	90.87	WP_211935310.1
C. telavivensis	93.42	WP_152400824.1
C. tructae	94.06	WP_135323790.1
C. werkmanii	91.72	WP_200009939.1
C. youngae	94.27	MBE1400439.1
Edwardsiella		
E. hoshinae	89.60	STC85157.1
E. piscicida	88.96	WP_225857556.1
E. tarda	89.81	WP_005289460.1
Escherichia		
E. albertii B156	99.36	OSL28878.1
E. fergusonii	100.00	EHT2456138.1
E. marmotae	99.36	WP_181474381.1
Grimontia		
G. celer	84.29	WP_062664976.1
G. hollisae	83.65	WP_005502395.1
G. indica	83.86	WP_002541160.1
G. marina	83.86	WP_062711725.1
G. sedimenti	84.08	WP_165018039.1

Haemophilus		
H. haemolyticus	85.71	MBS6047811.1
H. influenzae	86.41	WP_105876691.1
H. parainfluenzae	86.41	WP_197560834.1
Kluyvera		
K. ascorbata	91.51	WP_123650583.1
K. cryocrescens	94.26	VFS63491.1
K. georgiana	92.14	WP_064543156.1
Mannheimia		
M. bovis	88.96	WP_188157104.1
M. massilioguelmaensis	88.96	WP_044470443.1
Pantoea		
P. alhagi	95.54	WP_085070752.1
P. allii	91.08	WP_172898144.1
Pasteurella		
P. atlantica	86.20	WP_211597003.1
P. bettyae	88.75	WP_005759789.1
P. caecimuris	87.05	WP_135969291.1
P. dagmatis	89.38	WP_005763873.1
P. multocida	89.60	WP_126374005.1
P. oralis	88.75	WP_101775664.1
P. skyensis	85.35	WP_090922465.1
P. testudinis	81.53	WP_084256634.1
Photobacterium		
P. alginatilyticum	84.93	WP_160653036.1
P. frigidiphilum	83.65	WP_107241454.1
P. lipolyticum	84.50	WP_107285444.1
Rodentibacter		
R. heylii	86.84	WP_164028172.1
R. mrazii	87.05	WP_077494533.1
R. pneumotropicus	86.62	WP_077584526.1
R. ratti	86.84	WP_077497412.1
***Salmonella enterica* subsp.**		
S. Ball	91.93	EDV5023983.1
S. Bareilly	90.02	EDD0074088.1
S. Berkeley	94.06	EDR6298474.1
S. Eastbourne	88.96	ECA1898161.1
S. Typhimurium	100.00	EBW6030708.1
S. Virchow	100.00	EED6435280.1
S. salamae	91.51	ECI4646655.1
Shigella		
S. boydii	99.54	EAB7887218.1
S. dysenteriae	99.76	EFQ0036493.1
S. sonnei	100.00	EFZ5471173.1

Vibrio		
V. aerogenes	82.17	WP_073604739.1
V. alginolyticus	83.65	WP_086049229.1
V. orientalis	83.23	WP_004412794.1
V. owensii	84.50	WP_038894112.1
V. panuliri	83.44	WP_075706001.1
V. parahaemolyticus	84.50	EHA1071077.1
V. penaeicida	82.59	WP_126607768.1
V. ziniensis	84.29	WP_165310521.1
V.nales bacterium SWAT-3	83.44	EDK26603.1
others		
Alginatibacterium sediminis	82.17	WP_120353282.1
Buttiauxella noackiae	91.72	MCA1920741.1
Canicola haemoglobinophilus	89.81	WP_078219213.1
Endozoicomonas ascidiicola	83.44	WP_067587072.1
Frederiksenia canicola	88.32	WP_123956402.1
Gallibacterium genomosp	84.93	WP_065237692.1
Histophilus somni	87.69	WP_075293819.1
Leclercia adecarboxylata	91.85	HBH14678.1
Mesocricetibacter intestinalis	88.75	WP_133543425.1
Otariodibacter oris	87.69	WP_121123337.1
Pelistega suis	84.93	WP_171680966.1
Phocoenobacter uteri	86.84	WP_115315014.1
Serratia oryzae	91.08	WP_076942015.1
Ursidibacter maritimus	89.60	WP_157402367.1
Yokenella regensburgei	85.99	WP_038258145.1

SUPPLEMENTARY TABLE 3 Bacterial species with homologs of both TnaA and AbiS

**Species name**	**Scientific Name**	**SdiA Per. Ident (%)**	**TnaA Per. Ident (%)**
Acinetobacter	*Acinetobacter piscicola*	47.28	52.45
	Acinetobacter pittii	96.17	100.00
Aeromonas	*Aeromonas allosaccharophila*	47.64	52.88
	Aeromonas aquatica	46.26	84.50
	Aeromonas bestiarum	46.26	52.88
	Aeromonas caviae	47.12	84.93
	Aeromonas dhakensis	48.30	53.62
	Aeromonas diversa	45.55	82.80
	Aeromonas encheleia	46.60	84.29
	Aeromonas enteropelogenes	47.96	84.50
	Aeromonas eucrenophila	46.26	84.93
	Aeromonas finlandensis	46.28	53.30
	Aeromonas hydrophila	48.64	85.35
	Aeromonas jandaei	45.61	53.09
	Aeromonas lusitana	46.94	86.93
	Aeromonas media	47.62	85.35
	Aeromonas popoffii	46.94	53.52
	Aeromonas rivipollensis	46.60	85.35
	Aeromonas salmonicida	45.89	52.67
	Aeromonas sanarellii	47.64	85.74
	Aeromonas sobria	46.96	53.52
	Aeromonas tecta	45.61	84.93
	Aeromonas veronii	46.08	53.52
Chromobacterium	*Chromobacterium sphagni*	45.42	53.22
	Chromobacterium subtsugae	44.98	53.22
	Chromobacterium vaccinii	47.46	54.51
	Chromobacterium violaceum	46.10	53.43
Enterovibrio	*Enterovibrio baiacu*	48.11	57.69
	Enterovibrio calviensis	47.17	57.69
	Enterovibrio nigricans	47.40	83.01
	Enterovibrio norvegicus	47.67	57.26
Grimontia	*Grimontia celer*	50.00	84.29
	Grimontia indica	48.96	83.86
	Grimontia marina	47.46	83.86
	Grimontia sedimenti	48.96	84.08
Photobacterium	*Photobacterium chitinilyticum*	48.99	56.72
	Photobacterium frigidiphilum	46.13	83.65
	Photobacterium ganghwense	48.33	55.15
	Photobacterium indicum	46.13	57.57

	Photobacterium lipolyticum	48.49	84.50
	Photobacterium marinum	48.83	53.86
	Photobacterium profundum	46.45	83.65
	Photobacterium proteolyticum	49.16	84.71
Vibrio	*Vibrio cholerae*	50.67	85.53
	Vibrio parahaemolyticus	47.56	84.50

231

SUPPLEMENTARY TABLE 4 List of genes differentially expressed in the *abiS* mutant compared to the wild-type strain (Log_2-fold change ≥ 1.0). Significantly differentially expressed genes were determined by Cufflinks after Benjamini-Hochberg correction. The fold-change is the ratio of the mutant FPKM to the wild-type FPKM.

Class	Gene ID[a]	Log_2-fold change	Description
Amino acid transport and metabolism			
	A1S_0926	-2.71	choline dehydrogenase
	A1S_3402	-2.62	arginase/agmatinase/formimionoglutamate hydrolase
	A1S_0925	-2.60	choline dehydrogenase
	A1S_3404	-2.22	amino acid APC transporter
	A1S_3416	-2.15	glyoxalase/bleomycin resistance protein/dioxygenase
	A1S_2103	-2.05	transport protein
	A1S_1883	-1.99	3-dehydroquinate dehydratase
	A1S_3405	-1.88	histidine ammonia-lyase
	A1S_3418	-1.78	4-hydroxyphenylpyruvate dioxygenase
	A1S_3413	-1.57	APC family aromatic amino acid transporter
	A1S_1075	-1.49	D-amino-acid dehydrogenase
	A1S_1229	-1.43	pyrroline-5-carboxylate reductase
	A1S_1491	-1.34	glutamate/aspartate transport protein
	A1S_3133	-1.28	bifunctional N-succinyldiaminopimelate-aminotransferase/acetylornithine transaminase protein
	A1S_0924	-1.21	choline dehydrogenase
	A1S_0737	-1.16	5-methyltetrahydropteroyltriglutamate/homocysteine S-methyltransferase
	A1S_3406	-1.14	urocanate hydratase

	A1S_3131	-1.07	arginine succinyltransferase
	A1S_3407	-1.07	urocanase
	A1S_1372	-1.05	hypothetical protein
	A1S_1490	-1.00	glutamate/aspartate transport protein
	A1S_2449	-1.00	aromatic amino acid APC transporter
	A1S_0849	1.01	tartrate dehydrogenase
	A1S_2136	1.06	glutamine synthetase
	A1S_2351	1.07	glutamine synthetase
	A1S_2352	1.07	glutamine synthetase
	A1S_1390	1.51	hypothetical protein
Carbohydrate transport and metabolism			
	A1S_1888	-2.33	transport protein
	A1S_1331	-2.20	major facilitator superfamily transporter
	A1S_1210	-2.17	major facilitator superfamily transporter
	A1S_1880	-1.44	pyrroloquinoline-quinone QuiA
	A1S_0722	-1.24	major facilitator superfamily cis%2Ccis-muconate transporter
	A1S_0108	-1.16	major facilitator superfamily metabolite/H(+) symporter
	A1S_0486	-1.11	thermoresistant gluconokinase
	A1S_2057	-1.07	major facilitator superfamily methyl viologen resistance protein
	A1S_0803	-1.06	trehalose-6-phosphate synthase
	A1S_3270	1.04	permease
Cell envelope biogenesis outer membrane			
	A1S_1881	-2.03	porin
	A1S_0059	-1.12	glycosyltransferase
	A1S_0058	-1.04	glycosyltransferase
	A1S_0043	1.04	hypothetical protein

	A1S_1508	1.08	fimbrial biogenesis outer membrane usher protein
	A1S_3363	1.55	membrane metalloendopeptidases proteins
Cell motility and secretion			
	A1S_2215	-1.48	protein CsuC
	A1S_1508	1.08	fimbrial biogenesis outer membrane usher protein
Cell wall			
	A1S_0051	-1.99	outer membrane protein
Coenzyme metabolism			
	A1S_1698	-2.16	lipoyl synthase
	A1S_1213	-1.75	benzoate 12-dioxygenase electron transfer component
	A1S_1356	-1.21	4-hydroxybenzoate 3-monooxygenase
	A1S_3391	-1.20	thiamin-monophosphate kinase
	A1S_1995	-1.09	molybdopterin biosynthesis protein
	A1S_1345	-1.02	hypothetical protein
	A1S_1346	-1.02	phenylacetyl-CoA ligase
	A1S_2397	1.02	hypothetical protein
Defense mechanism			
	A1S_1800	1.06	RND efflux transporter
DNA replication recombination			
	A1S_3104	-1.75	ATP-dependent RNA helicase
	A1S_0648	1.00	hypothetical protein
	A1S_1749	1.05	ABC transporter-like protein
Energy production and conversion			
	A1S_1528	-2.69	bifunctional proline dehydrogenase/pyrroline-5-carboxylate dehydrogenase
	A1S_1700	-2.40	acetoin:26-dichlorophenolindophenol oxidoreductase subunit beta

	A1S_0927	-2.11	betaine aldehyde dehydrogenase
	A1S_1089	-2.01	hypothetical protein
	A1S_2098	-1.98	alcohol dehydrogenase
	A1S_2102	-1.47	aldehyde dehydrogenase 1
	A1S_2196	-1.36	membrane-associated dicarboxylate transport protein
	A1S_0723	-1.22	L-carnitine dehydrogenase
	A1S_1986	-1.13	fumarate hydratase
	A1S_0149	1.01	membrane-bound ATP synthase F0 sector%2C subunit a
	A1S_1905	1.06	24-dienoyl-CoA reductase
	A1S_0566	1.10	pyridine nucleotide transhydrogenase (proton pump) subunit alpha (part1)
	A1S_2005	1.16	nitrite reductase
	A1S_1857	1.36	vanillate O-demethylase oxidoreductase
Inorganic ion transport and metabolism			
	A1S_1882	-2.92	3-dehydroshikimate dehydratase
	A1S_2139	-2.06	hypothetical protein
	A1S_2140	-2.03	potassium-transporting ATPase subunit B
	A1S_2142	-1.73	hypothetical protein
	A1S_2141	-1.40	potassium-transporting ATPase subunit A
	A1S_0977	1.06	arylsulfatase
	A1S_2077	1.16	outer membrane porin receptor for Fe(III)-coprogen%2C Fe(III)-ferrioxamine B and Fe(III)-rhodotrulic acid uptake (fhuE)
	A1S_2070	1.37	magnesium-transporting ATPase MgtA
	A1S_0947	1.47	vanillate O-demethylase oxygenase subunit (VanA-like)
	A1S_1861	1.48	benzoate dioxygenase large subunit
	A1S_1860	1.57	ring hydroxylating dioxygenase Rieske (2Fe-2S) protein
	A1S_0170	1.61	outer membrane copper receptor (OprC)

	A1S_2748	1.73	ammonium transporter
Intracellular trafficking and secretion			
	A1S_1303	-1.11	hypothetical protein
	A1S_0454	1.55	biopolymer transport protein (ExbD)
Lipid metabolism			
	A1S_2099	-2.80	hypothetical protein
	A1S_1109	-2.36	feruloyl-CoA synthase
	A1S_1849	-1.81	beta-ketoadipyl CoA thiolase
	A1S_1891	-1.73	beta-ketoadipyl CoA thiolase
	A1S_1894	-1.58	3-oxoacid CoA-transferase subunit A
	A1S_1846	-1.49	3-oxoacid CoA-transferase subunit A
	A1S_0106	-1.48	enoyl-CoA hydratase/isomerase
	A1S_1111	-1.27	p-hydroxycinnamoyl CoA hydratase/lyase
	A1S_1373	-1.24	acyl-CoA carboxylase subunit alpha protein
	A1S_1374	-1.21	3-methylglutaconyl-CoA hydratase
	A1S_1497	-1.16	acyltransferase
	A1S_0522	-1.14	3-oxoacyl-ACP synthase
	A1S_0105	-1.12	acyl-CoA dehydrogenase
	A1S_1121	-1.08	lipase/esterase
	A1S_1108	-1.07	acyl-CoA dehydrogenase
	A1S_1375	-1.02	propionyl-CoA carboxylase (Beta subunit)
	A1S_1277	1.01	allophanate hydrolase subunit 2
	A1S_1790	1.11	6-phosphogluconate dehydrogenase
Nucleotide transport and metabolism			
	A1S_1890	-1.58	3-carboxy-cis%2Ccis-muconate cycloisomerase
	A1S_2586	-1.29	deoxyguanosinetriphosphate triphosphohydrolase-like protein

	A1S_1504	-1.20	purine-cytosine permease
	A1S_3082	1.02	glutamine amidotransferase
Posttranslational modification			
	A1S_3415	-1.54	maleylacetoacetate isomerase
	A1S_0135	-1.02	hypothetical protein
	A1S_0011	1.16	hypothetical protein
	A1S_1460	1.19	alkyl hydroperoxide reductase subunit F
	A1S_1201	1.33	alkyl hydroperoxide reductase subunit F
	A1S_1458	1.44	alkyl hydroperoxide reductase subunit F
Secondary metabolites biosynthesis transport			
	A1S_1884	-2.94	protocatechuate 34-dioxygenase subunit alpha
	A1S_1885	-2.71	protocatechuate 34-dioxygenase subunit beta
	A1S_3403	-2.51	imidazolonepropionase
	A1S_1697	-1.99	transcriptional regulator
	A1S_1211	-1.93	benzoate transporter
	A1S_1212	-1.68	1%2C6-dihydroxycyclohexa-2%2C4-diene-1-carboxylate dehydrogenase
	A1S_1214	-1.49	benzoate 12-dioxygenase subunit beta
	A1S_1704	-1.39	acetoin dehydrogenase
	A1S_1845	-1.26	CatA3
	A1S_3414	-1.18	fumarylacetoacetase
	A1S_1844	-1.04	CatC3
	A1S_0907	1.07	hypothetical protein
	A1S_1854	1.09	tyramine oxidase
	A1S_0948	1.11	3-ketoacyl-ACP reductase

	A1S_3009	1.11	short chain dehydrogenase/reductase family oxidoreductase
	A1S_2390	1.13	acinetobactin biosynthesis protein
	A1S_1858	1.87	short-chain dehydrogenase
	A1S_1859	1.87	aromatic-ring-hydroxylating dioxygenase subunit beta
Signal transduction mechanisms			
	A1S_2138	-1.31	hypothetical protein
	A1S_0109	-1.10	homoserine lactone synthase
	A1S_2137	-1.06	hypothetical protein
Transcription			
	A1S_2235	-2.02	regulatory protein LysR
	A1S_0928	-1.63	transcriptional regulator BetI
	A1S_1074	-1.43	hypothetical protein
	A1S_1738	-1.43	transcriptional regulator
	A1S_1216	-1.09	LysR regulator
	A1S_2152	-1.03	AraC family transcriptional regulator
	A1S_1228	-1.02	cold shock protein
	A1S_1047	1.03	chromosome replication initiation inhibitor protein
	A1S_1419	1.04	anti-sigm factor ChrR
	A1S_0253	1.05	transcriptional regulator
	A1S_2463	1.21	ribosomal large subunit pseudouridine synthase A(RluA-like)
	A1S_1232	1.25	EsvB
	A1S_1256	1.35	transcriptional regulator
	A1S_2295	1.36	transcriptional regulator
Translation ribosomal structure and biogenesis			
	A1S_0827	-1.41	peptidyl-tRNA hydrolase
	A1S_3067	-1.15	30S ribosomal protein S8

	A1S_1527	-1.12	tRNA (uracil-5-)-methyltransferase
	A1S_2423	1.19	50S ribosomal protein L31
	A1S_0360	1.32	30S ribosomal protein S15
	A1S_1865	1.86	Glu-tRNA amidotransferase
Hypothetical protein and others			
	A1S_2218	-2.57	protein CsuA/B
	A1S_1383	-2.56	surface antigen
	A1S_1209	-2.22	benzoate transport porin (BenP)
	A1S_1889	-2.12	3-oxoadipate enol-lactonase
	A1S_0996	-2.05	hypothetical protein
	A1S_1887	-2.01	4-hydroxybenzoate transporter
	A1S_1850	-1.95	esterase
	A1S_1886	-1.81	gamma-carboxymuconolactone decarboxylase (CMD)
	A1S_0931	-1.67	BCCT family high-affinity choline transporter
	A1S_1077	-1.55	hypothetical protein
	A1S_2843	-1.51	hypothetical protein
	A1S_0333	-1.51	tRNA-Met
	A1S_1380	-1.49	hypothetical protein
	A1S_2100	-1.38	hypothetical protein
	A1S_2162	-1.30	hypothetical protein
	A1S_1385	-1.29	hypothetical protein
	A1S_1760	-1.27	hypothetical protein
	A1S_3144	-1.25	hypothetical protein
	A1S_2151	-1.24	AraC family transcriptional regulator
	A1S_1384	-1.22	CinA-like protein
	A1S_2165	-1.22	hypothetical protein
	A1S_2513	-1.20	tRNA-Asn

A1S_1437	-1.17	acyl-CoA dehydrogenase
A1S_1661	-1.15	fructose-26-bisphosphatase
A1S_1122	-1.15	short-chain dehydrogenase
A1S_2982	-1.14	hypothetical protein
A1S_0550	-1.10	VGR-like protein
A1S_0830	-1.09	tRNA-Gln
A1S_2336	-1.08	hypothetical protein
A1S_3303	-1.06	hypothetical protein
A1S_0334	-1.06	hypothetical protein
A1S_0627	-1.05	hypothetical protein
A1S_2428	-1.04	ATP-dependent protease
A1S_2440	-1.02	hypothetical protein
A1S_2185	-1.01	hypothetical protein
A1S_0779	-1.01	hypothetical protein
A1S_2016	1.01	phage-related lysozyme
A1S_1709	1.02	hypothetical protein
A1S_1981	1.03	hypothetical protein
A1S_0635	1.04	hypothetical protein
A1S_3116	1.06	hypothetical protein
A1S_3277	1.06	pirin-like protein
A1S_2333	1.07	hypothetical protein
A1S_2020	1.07	hypothetical protein
A1S_0303	1.07	peptide signal
A1S_1599	1.09	hypothetical protein
A1S_2416	1.12	hypothetical protein
A1S_1901	1.16	hypothetical protein
A1S_1166	1.19	hypothetical protein
A1S_0871	1.19	metal-dependent hydrolase

A1S_0862	1.20	hypothetical protein
A1S_1042	1.20	hypothetical protein
A1S_2029	1.24	hypothetical protein
A1S_2021	1.27	hypothetical protein
A1S_3350	1.28	hypothetical protein
A1S_1856	1.29	p-hydroxyphenylacetate hydroxylase C1
A1S_2754	1.34	MFS family transporter
A1S_0946	1.40	hypothetical protein
A1S_3411	1.43	G3E family GTPase
A1S_1862	1.44	hypothetical protein
A1S_1932	1.46	hypothetical protein
A1S_0384	1.46	Zinc-binding protein
A1S_1933	1.48	hypothetical protein
A1S_2069	1.49	magesium transporter transmembrane protein
A1S_1863	1.64	hypothetical protein
A1S_2623	1.67	hypothetical protein
A1S_1864	1.78	acyl-CoA dehydrogenase-like protein
A1S_0169	1.85	hypothetical protein
A1S_1377	2.08	acrR family transcriptional regulator

a. Gene ID are from https*://www.ncbi.nlm.nih.gov/genome/403?genome_assembly_id=300108.* Annotations and functional assignments are from *Acinetobacter baumannii* ATCC17978*.*

SUPPLEMENTARY TABLE 5 Bacterial strains and plasmids used in this study

**Strain or plasmid** [a]	**Phenotype and/or characteristic(s)** [b]	**Source or reference**
A. baumannii		
ATCC17978	Wild-type strain of *A. baumannii*	Laboratory collection
Δ*abiS*	Indole-minus mutant derived from ATCC17978 with *abiS* being deleted	This study
Δ*abiS*(*abiS*)	Mutant *abiS* harboring the expression construct *pwh1266-abiS*	This study
ATCC17978(P*abaI-lacZ*)	ATCC17978 harboring the reporter construct P*abaI-lacZ*	This study
ΔabiS(P*abaI-lacZ*)	Δ*abiS* harboring the reporter construct P*abaI-lacZ*	This study
ATCC17978 (P*epsA*-*lacZ*)	ATCC17978 harboring the reporter construct P*epsA-lacZ*	This study
Δ*abiS*(P*epsA*-*lacZ*)	Δ*abiS* harboring the reporter construct P*epsA*-*lacZ*	This study
ATCC17978 (P*pgaA*-*lacZ*)	ATCC17978 harboring the reporter construct P*pgaA*-*lacZ*	This study
Δ*abiS*(P*pgaA*-*lacZ*)	Δ*abiS* harboring the reporter construct P*pgaA*-*lacZ*	This study
ATCC17978(P*plD-lacZ*)	ATCC17978 harboring the reporter construct P*plD-lacZ*	This study
Δ*abiS*(P*plD-lacZ*)	Δ*abiS* harboring the reporter construct P*plD-lacZ*	This study
ATCC17978 (pRSETB-*mCherry*)	ATCC17978 harboring the plasmid pRSETB-mCherry	This study
ΔabiS (pRSETB-*mCherry*)	Δ*abiS* harboring the plasmid pRSETB-mCherry	This study
Δ*abiS* (pRSETB-*abiS-mCherry*)	Δ*abiS* harboring the expression construct pRSETB-*abiS*-mCherry	This study
E. Coli		
DH5α	*supE44 lacU169(80lacZ M15) hsdR17 recA1 endA1 gyrA96 thi-1 relA1 pir*	Laboratory collection
K12		Laboratory collection
Δ*tnaA*	*K12* with *tnaA* being deleted	This study
Δ*tnaA(tnaA)*	Δ*tnaA* harboring the expression construct *pwh1266-tnaA*	This study
Δ*tnaA(abiS)*	Δ*tnaA* harboring the expression construct *pwh1266-abiS*	This study

P. aeruginosa		
PAO1	Wild-type strain of *P. aeruginosa*	Laboratory collection
PAO1 (pBBRI-MCS5-GFP)	PAO1 harboring the plasmid pBBRI -MCS5-GFP	Laboratory collection
PAO1 (P*lasI*-*lacZ*)	PAO1 harboring the reporter construct P*lasI*-*lacZ*	Laboratory collection
PAO1 (P*lasR*-*lacZ*)	PAO1 harboring the reporter construct P*lasR*-*lacZ*	Laboratory collection
PAO1 (P*rhlI*-*lacZ*)	PAO1 harboring the reporter construct P*rhlI*-*lacZ*	Laboratory collection
PAO1 (P*rhlR*-*lacZ*)	PAO1 harboring the reporter construct P*rhlR*-*lacZ*	Laboratory collection
PAO1 (P*pqsA*-*lacZ*)	PAO1 harboring the reporter construct P*pqsA*-*lacZ*	Laboratory collection
PAO1 (P*pqsR*-*lacZ*)	PAO1 harboring the reporter construct P*pqsR*-*lacZ*	Laboratory collection
PAO1 (P*exsCEBA*-*lacZ)*	PAO1 harboring the reporter construct P*exsCEBA*-*lacZ*	Laboratory collection
**Plasmid**		
pCasAb	pCasAb, Apr^r	(7)
pSGAb	pSGAb, Kan^r	(7)
pWH1266	Broad-host-range cloning vector, Amp^r	Laboratory collection
pK18	Kan^r	Laboratory collection
pWH1266- *abiS*	pWH1266 containing the *abiS*, Amp^r	This study
pWH1266- *tnaA*	pWH1266 containing the *tnaA*, Amp^r	This study
pME2-*lacZ*	Broad-host-range cloning vector, Tet^r	(8)
P*abaI*-*lacZ*	pME2-*lacZ* containing the promoter of *abaI* Tet^r	This study
P*epsA*-*lacZ*	pME2-*lacZ* containing the promoter of *epsA* Tet^r	This study
P*pgaA*-*LacZ*	pME2-*lacZ* containing the promoter of *pgaA*, Tet^r	This study
P*plD*-*LacZ*	pME2-*lacZ* containing the promoter of *plD*, Tet^r	This study
pRSETB-*mCherry*	pRSETB containing the gene of *mCherry*, Amp^r	Laboratory collection
pRSETB-*abiS*-*mCherry*	pRSETB-*mCherry* containing the *abiS*,	This study

Amp^{r}

[a] The bacterial strains and plasmids used in this study have been sequenced to ensure the correct construction.

[b] Apr^{r}, Kan^{r}, Amp^{r} and Trim^{r} indicate resistance to apramycin, kanamycin, ampicillin and tetracycline, respectively.

SUPPLEMENTARY TABLE 6 PCR primers used in this study

Primer	Sequence (5'-3')
For deletion	
pSGAb-F	GTCTGCTTCTTCCAGCCCTC
pSGAb-R	AGCGGATAACAATTTCACACAGG
pCasAb-F	AACTCGCCAAATCACTAAGCA
pCasAb-R	ACTGAATAAGCTACCGTTGGAC
abiS-spa-F	tagtGTTTTTAACCATGGTATGGC
abiS-spa-R	aaacGCCATACCATGGTTAAAAAC
abiS-ssDNA	GAAGCTGCTTCTCATACAAAATAAAAATAGTGAGTAAAAAGCTTTAAAACAAAAAATGCGCCAAAAGGCGCATTTTTTAC
abiS-out-F	TGGCAACAGCGAGTGACTGG
abiS-out-R	ATCGCTAAGAAAAACCGTGGTT
tnaA-L-F	tatgaccatgattacgaattcGGGGAATTTACTTCAGACGACCT
tnaA-L-R	ccactctgtagtattaaTACATAATCCTTCATTTATTTTAATTACAGTG
tnaA-R-F	gtaTTAATACTACAGAGTGGCTATAAGGATGTT
tnaA-R-R	acgacggccagtgccaagcttGAAAGAACCAAACACGATCACAAA
For *in trans* expression	
abiS-F	ccacacccgtcctgtggatccATGAAAAGGAACTTAATATTTTTCTGTGC
abiS-R	tgcgtccggcgtagaggatccTTAAAGGAACAGATGGTGGCG
tnaA-F	tgcgtccggcgtagaggatccATGGAAAACTTTAAACATCTCCCTG
tnaA-R	ccctttcgtcttcaagaattcTTAAACTTCTTTCAGTTTTGCGGT
For reporter	
P*abaI*-*lacZ*-F	cggtgagaatggcaaaagcttCGAATGAACGAAAACCAACTCA
P*abaI*-*lacZ*-R	taatcatggtcatagctcgagTGTTTTGAAATCCAGCAATAATATTCA
P*epsA*-*lacZ*-F	cggtgagaatggcaaaagcttCCAACTACCGGCCCTTTTTT
P*epsA*-*lacZ*-R	taatcatggtcatagctcgagCCGCACAGCTGGCAGCAC
P*pgaA*-*LacZ*-F	cggtgagaatggcaaaagcttCGTCGTATTGGTTAATTAAGTCAATTA
P*pgaA*-*LacZ*-R	taatcatggtcatagctcgagCCAATGAAAAAACACATTTCTTTAGC
P*plD*-*LacZ*-F	cggtgagaatggcaaaagcttCCAATCCAGTACGCACGAGAT
P*plD*-*LacZ*-R	taatcatggtcatagctcgagTACGTTTTAAGTCTAAAATCATATCCATCG
Others	

pWH1266- *tnaA*-F	ccacacccgtcctgtggatccATGGAAAACTTTAAACATCTCCCTG
pWH1266- *tnaA*-R	tgcgtccggcgtagaggatccTTAAACTTCTTTAAGTTTTGCGGTG
P*abiS*-mCherry-F	cgacgatgacgataaggatccATGAAAAGGAACTTAATATTTTTCTGTGC
P*abiS*-mCherry-R	cgacgatgacgataaggatccATGAAAAGGAACTTAATATTTTTCTGTGC
For RT-qPCR	
abaI- F	GTTGTGCCAGACTACTACCCA
abaI- R	CAGCCTGACTGCTAGAGGAA
epsA- F	TGTGATGGGTGAGTCAGGTA
epsA- R	AAGCATTTGAGAACGGGATA
pgaA- F	GAGTCCGTCTTGATTGGTCC
pgaA- R	AGCTGTTTGATCTTGGCTGT
plD- F	AGTTCTGAACGGTGGCTTGT
plD- R	GATGTCATGTTGGTTGGTGC
ATCC17978-16S-F	ACTTCGTAAGGAATAAATCCCC
ATCC17978-16S-R	TGTGGCTCATCCTTATGCTGA
lasI-F	CCGTTTCGCCATCAACTCTG
lasI-R	GATCATCATCTTCTCCACGCCTA
rhlI-F	CCATCCGCAAACCCGCTACA
rhlI-R	TCACCGCCACCACCGAACTG
pqsA-F	CAAGGTGAATGGCCGCTGGGTG
pqsA-F	CGGAAGGTTGTCGTGGTAGAGGGTGT
lasR-F	GCCTTCATCGTCGGCAACTAC
lasR-R	GCGCACCACTGCAACACTTC
rhLR-F	CTCCTCGGAAATGGTGGTCTGG
rhLR-R	CGGAAAGCACGCTGAGCAAAT
pqsR-F	CCTGCGGGTGCTGCTGGATA
pqsR-R	CGACGACGAACGCCTTGGTGTAG
exsA-F	GGCGGCGATAGCTCTGGGTGAAAT
exsA-R	CGCCGCGGAAGCTATGTCGTAAGT
exsC-F	TGGATTTAACGAGCAAGGTCAA
exsC-R	CGAGAATCTGCGCATACAACTG
PAO1-16S-F	GCGCAACCCTTGTCCTTAGTT
PAO1-16S-R	TGTCACCGGCAGTCTCCTTAG

Underlined sequences: Protective base and restriction endonuclease recognition sequences

REFERENCES

1 Qi L, Li H, Zhang C, Liang B, Li J, Wang L, Du X, Liu X, Qiu S, Song H . 2016. Relationship between antibiotic resistance, biofilm formation, and biofilm-specific resistance in *Acinetobacter baumannii.* Front Microbiol 7:483.

2 Lai Y, Liu CW, Chi L, Ru H, Lu K. 2021. High-Resolution Metabolomics of 50 Neurotransmitters and Tryptophan Metabolites in Feces, Serum, and Brain Tissues Using UHPLC-ESI-Q Exactive Mass Spectrometry. ACS omega 6: 8094–8103.

3 Livak KJ, Schmittgen TD. 2001. Analysis of relative gene expression data using real-time quantitative PCR and the 2(-Delta Delta C(T)) Method. Methods 25:402-408.

4 Cui C, Yang C, Song S, Fu S, Sun X, Yang L, He F, Zhang LH, Zhang Y, Deng Y. 2018. A novel two-component system modulates quorum sensing and pathogenicity in *Burkholderia cenocepacia.* Mol Microbiol 108:32-44.

5 Langmead B, Salzberg SL. 2012. Fast gapped-read alignment with Bowtie 2. Nat Methods 9:357-359.

6 Trapnell C, Williams BA, Pertea G, Mortazavi A, Kwan G, van Baren MJ, Salzberg SL, Wold BJ, Pachter L. 2010. Transcript assembly and quantification by RNA-Seq reveals unannotated transcripts and isoform switching during cell differentiation. Nat Biotechnol 28:511-515.

7 Wang Y, Wang Z, Chen Y, Hua X, Yu Y, Ji Q. 2019. A highly efficient CRISPR-Cas9-based genome engineering platform in *Acinetobacter baumannii* to understand the H_2O_2-sensing mechanism of OxyR. Cell Chem Biol 26:1732-1742.

8 Kovach ME, Elzer PH, Hill DS, Robertson GT, Farris MA, Roop RM 2nd, Peterson KM. 1995. Four new derivatives of the broad-host-range cloning vector pBBR1MCS, carrying different antibiotic-resistance cassettes. Gene 166:175-176.
